# Tissue-type plasminogen activator exerts EGF-like chemokinetic effects on oligodendrocytes in white matter (re)myelination

**DOI:** 10.1186/s13024-017-0160-5

**Published:** 2017-02-23

**Authors:** Camille Leonetti, Richard Macrez, Mathilde Pruvost, Yannick Hommet, Jérémie Bronsard, Antoine Fournier, Maxime Perrigault, Isabel Machin, Denis Vivien, Diego Clemente, Fernando De Castro, Eric Maubert, Fabian Docagne

**Affiliations:** 1 Normandie Univ, UNICAEN, INSERM U1237, Physiology and imaging of neurological disorders (PhIND), Cyceron, Caen, 14000 France; 2 Grupo de Neurobiología del Desarrollo-GNDe, Hospital Nacional de Parapléjicos-SESCAM, Toledo, Spain; 3 Grupo de Neuroinmuno-reparación, Hospital Nacional de Parapléjicos, Toledo, Spain; 4 Grupo de Neurobiología del Desarrollo (GNDe), Instituto Cajal, CSIC, Madrid, Spain; 5 Inserm, Centre Cyceron, Bvd Becquerel, BP5229, Caen Cedex, 14074 France

**Keywords:** Multiple sclerosis, Myelin, Epidermal growth factor, Endothelial cells, Vasophilic migration, Lysolecithin, Spinal cord, Development

## Abstract

**Background:**

The ability of oligodendrocyte progenitor cells (OPCs) to give raise to myelin forming cells during developmental myelination, normal adult physiology and post-lesion remyelination in white matter depends on factors which govern their proliferation, migration and differentiation. Tissue plasminogen activator (tPA) is a serine protease expressed in the central nervous system (CNS), where it regulates cell fate. In particular, tPA has been reported to protect oligodendrocytes from apoptosis and to facilitate the migration of neurons. Here, we investigated whether tPA can also participate in the migration of OPCs during CNS development and during remyelination after focal white matter lesion.

**Methods:**

OPC migration was estimated by immunohistological analysis in spinal cord and corpus callosum during development in mice embryos (E13 to P0) and after white matter lesion induced by the stereotactic injection of lysolecithin in adult mice (1 to 21 days post injection). Migration was compared in these conditions between wild type and tPA knock-out animals. The action of tPA was further investigated in an in vitro chemokinesis assay.

**Results:**

OPC migration along vessels is delayed in tPA knock-out mice during development and during remyelination. tPA enhances OPC migration via an effect dependent on the activation of epidermal growth factor receptor.

**Conclusion:**

Endogenous tPA facilitates the migration of OPCs during development and during remyelination after white matter lesion by the virtue of its epidermal growth factor-like domain.

**Electronic supplementary material:**

The online version of this article (doi:10.1186/s13024-017-0160-5) contains supplementary material, which is available to authorized users.

## Background

Spontaneous remyelination enables regeneration of white matter after lesions. This process is possible thanks to the presence, in the adult central nervous system (CNS), of oligodendrocyte precursor cells (OPCs) which have the capacity to replace dead oligodendrocytes and to form new myelin sheaths at the sites of demyelination [1]. Spontaneous remyelination is linked to the ability of OPCs to proliferate, migrate and differentiate into mature oligodendrocytes in physiological conditions (during development and in adulthood) and in response to injuries.

This process is important in various pathological situations in the CNS, including multiple sclerosis (MS). In relapsing-remitting MS (RR-MS), the most frequent form of the disease, patients experience successive phases of relapses (worsening of symptoms) and remissions (neurological improvement), which are considered to mostly follow a pattern of successive demyelination and remyelination, giving rise to the so-called ‘shadow plaques’. However, the extent of remyelination is very variable among patients, and, in most cases, incomplete [2, 3]. A second form of MS exists, called primary progressive MS (PP-MS), in which the disease progresses gradually without remission phases. Finally, in a significant subset of RR-MS patients, the course of the disease eventually shifts to a progressive course, termed secondary progressive MS (SP-MS). In these progressive forms of MS (PP-MS and SP-MS), although partial remyelination has been observed [4], its efficiency is not sufficient for counterbalance the progression of disease. Therefore, there is great interest in understanding the endogenous factors which govern spontaneous remyelination in order to explain why this process fails in the different forms of MS. This better knowledge could help designing new therapeutic strategies aiming at boosting the capacity of remyelination in MS and other demyelinating diseases [5, 6].

Most remyelinating oligodendrocytes are derived from adult OPCs, which have a widespread distribution throughout the CNS [7]. Effective remyelination depends in great part on the ability of these OPCs to reach the demyelinated areas. Migration of OPCs depends on the action of chemotactic factors able to attract or repulse OPCs, thus controlling the direction of migration, and chemokinetic factors able to accelerate or slowdown OPCs, thus controlling the speed of migration [8]. Although embryonic OPCs, responsible for myelination during development, and adult OPCs, responsible for remyelination after lesion, form two distinct populations, developmental myelination and post-lesion remyelination share mechanistic aspects [9, 10]. For this reason, lessons learned from the description of myelination during development can be useful to better understand the chemotactic and chemokinetic factors involved in remyelination.

Tissue-type plasminogen activator (tPA) is a serine protease long described for its role in fibrinolysis in the circulation. Beyond this property, tPA has been reported to be expressed by many cell types in the brain, including oligodendrocytes [11, 12] and to exert multiple functions in the healthy and diseased CNS [13]. Among these functions, tPA has been shown to facilitate neuron migration during development [14]. tPA has also been shown to influence neuron and OPC survival, in particular by exerting anti-apoptotic effects on these cells via the action of a structural domain homologous to epidermal growth factor (EGF) [11, 15], a growth factor previously described to increase OPC migration [16]. However, whether tPA could influence OPC migration was never investigated so far.

In the present study, we hypothesized that tPA could facilitate OPC migration based on the following facts described above: (i) tPA facilitates neuronal migration [14], (ii) tPA can act on OPCs through its EGF-like domain [11] and (iii) EGF increases OPC migration [16]. To study this, we first explored the migration of OPCs in the developing spinal cord and telencephalon (giving raise to corpus callosum, the vastest white matter area in murine brain) by comparing wild type (WT) and tPA knock-out (tPA^−/−^) animals. Because we observed a delay in OPC migration during myelination, we studied whether a similar phenotype could appear in adult tPA^−/−^ mice after focal white matter lesion. Indeed, remyelination was also delayed in these animals, indicating that endogenous tPA could facilitate OPC migration. Finally, we show here that tPA exerts a chemokinetic effect on OPCs in vitro, through its domain of homology with EGF.

## Methods

### Ethical statement

Experiments were performed in our laboratory (agreement number D14118001) in accordance with French ethical laws (act no. 87–848; *Ministère de l’Agriculture et de la Forêt*), European Union Council Directives of November 24, 1986 (2010/63/EEC) and guidelines for the care and use of laboratory animals. Experiments have been approved by the ethics committee n°52 on animal experiments (CENOMEXA) and by the French Ministry of Research under the project license number 02653.2 (30/06/2016). None of the experimental procedures induced animal mortality. All experiments were performed following the ARRIVE guidelines (www.nc3rs.org.uk), including randomization of treatment as well as analysis blind to the treatment.

### Animals and surgery

Wild type and tPA^−/−^ [17] C57BL6-J mice (aged 2 months, CURB, Caen, France) were housed in a temperature-controlled room on a 12 h light/12 h dark cycle with access to food and water ad libitum. Demyelination was induced by stereotaxic injection of 1% LPC (L1381, Sigma-Aldrich) in 0.1 M phosphate-buffered saline (Sigma-Aldrich). Mice were deeply anaesthetized with isoflurane (induction 5%, maintenance 2.5% in 70/30% NO_2_/O_2_). Rectal temperature was maintained at 37 °C using a feedback-regulated heating pad. Mice were placed in a stereotaxic frame. The demyelinating agent was injected unilaterally into the corpus callosum with a microinjection pipette (Hecht Assistent), using the following stereotaxic coordinates: 1.1 mm anterior to the bregma, 2.7 mm lateral to the bregma and 1.3 mm deep from the cortex surface, using an angle of 30°. Scalp incisions were closed with Vetbond thread.

### Magnetic resonance imaging (MRI) analysis

MRI analyses were performed on 1, 3, 7, 14 and 21 days post-injection. Experiments were carried out on a Pharmascan 7 T MRI (Bruker, Germany). T2-weighted images were acquired using a multislice/multiecho sequence: TE/TR 12.7 ms/2500 ms and a flip angle of 180° with spatial resolution of 70 μm*70 μm and slice thickness 350 μm interpolated to an isotropic resolution of 70 μm (acquisition time = 8 min). Lesion sizes were quantified on these images by an experimenter blinded to the genotype using ImageJ (NIH software v1.49e, National Institute of Health, Bethesda, MD, USA).

### Immunohistochemistry

1, 3, 7, 14 and 21 days post-injection mice were deeply anesthetized and perfused transcardially with 20 ml of cold heparinized NaCl 0.9%, followed by 2% paraformaldehyde and 0.2% picric acid in 150 ml of 0.1 M sodium phosphate buffer, pH 7.4. Brains were removed, washed in veronal buffer containing 20% sucrose, and frozen in Tissue-Tek (Miles Scientific). Embryos were collected at noon of embryonic days. Heads and trunks were fixed in 2% paraformaldehyde and 0.2% picric acid for 24 h, cryoprotected in veronal buffer containing 20% sucrose, and frozen in Tissue-Tek. Cryomicrotome-cut sections (10 μm) were collected on poly-lysine slides and stored at −80 °C before processing. Sections were incubated overnight at room temperature with a primary antibody or a cocktail of primary antibodies (Additional file 1: Table S1) diluted in veronal buffer containing 0.25% Triton ×100. Three rinses were performed in veronal buffer at room temperature. All secondary antibodies (Additional file 1: Table S1) were diluted (1:600) in veronal buffer containing 0.25% Triton × 100. Incubation was performed at room temperature for 1h30min followed by three washes. Washed sections were coverslipped with antifade medium containing DAPI. For each set of immunostaining, the following controls were systematically performed on adjacent sections: Omission of primary or secondary antibodies in single immunolabelling experiments resulted in no specific labelling. Additionally, the absence of cross-reactivity between the antibodies in multi-immunolabelling experiments was demonstrated by omission of one of the primary antibodies and consecutively the absence of relevant signal detection with the cocktail of secondary antibodies.

Images were digitally captured using a Leica DM6000B microscope-coupled coolsnap camera (ORCA Flash 4-LT; Hamamatsu), visualized with Metamorph 5.0 software (Molecular Devices) and further processed using ImageJ1.5 h software (NIH).

### 3D reconstruction

Images were collected using a Leica SP5 confocal microscope with a 100× oil-immersive objective (Leica Microsystems). Confocal images were taken at a 1024 × 1024 pixel resolution with a z-step of 0.45 μm. The 3D structure was reconstructed from confocal images using Imaris software (version 5.5, Bitplane, Zurich, Switzerland). Volume and surface functions were used.

### Cell counting and quantification of MBP immunofluoresence

The total number of positive cells was counted in the lesion area. In the contralateral corpus callosum, an equivalent area for each mice was selected and the number of positive cells was counted in the selected area. During development, the area of the whole spinal cord or telencephalon section was measured and the total number of positive cells was counted in the section. For MBP immunofluorescence quantification, the mean gray value was evaluated in the lesion area or the equivalent contralateral area. All quantitative analyses were done in three randomly selected sections per mouse and the individual values for the number of cells/mm^2^ or for fluorescence intensity for each mouse was estimated by averaging the values of all counted sections for the same mouse.

### Oligodendrocyte precursor cell (OPC) cultures

Primary mixed glial cultures were prepared according to the modified technique of McCarthy and de Vellis [18]. Briefly, forebrains of P_0_-P_1_ newborn Wistar rats were dissociated mechanically and resuspended in DMEM (D5671, Sigma-Aldrich) containing 10% fetal bovine serum, 10% horse serum, 2 mM glutamine (25030024, Thermo Fisher Scientific), 0.5% penicillin streptomycin (15140122, Thermo Fisher Scientific) and plated on poly-D-lysine-coated (0.1 mg/mL) (P6407, Sigma-Aldrich) 75 cm^2^ flasks (Nunc, Wiesbaden, Germany). After 10 days in culture the flasks were shaken at 210 rpm at 37 °C for 3 h to remove loosely adherent microglia. The remaining OPCs present on the top of the confluent monolayer of astrocytes were dislodged by shaking overnight at 270 rpm. The cell suspension was filtered through a 40 μm nylon mesh and then pre-plated on bacterial grade Petri dishes for 1 h. The nonadherent OPCs that remained in suspension were recovered, filtered through a 40 μm nylon mesh, and plated again on bacterial grade Petri dishes for 30 min. The resulting enriched OPCs cell suspension was counted and seeded in accordance to the assay performed.

### Cell proliferation assay

OPC proliferation was estimated by examination of the cultures under bright-field microscopy and quantified with WST-1 assay. Briefly, oligodendrocyte precursor cell were plated on poly-D-lysine-coated (0.1 mg/mL), 24-well (2 cm^2^/well) tissue culture dish at a density of 6 × 10^4^ cells/cm^2^ and cultured in DMEM (D5671, Sigma-Aldrich) supplemented with recombinant PDGF-AA (PHG0035, Thermo Fisher Scientific, 5 ng/ml) and bFGF (15140–122, Invitrogen, 5 ng/ml), N2 supplement (17502–048, Thermo Fisher Scientific), 2 mM glutamine (25030024, Thermo Fisher Scientific), 0.5% penicillin streptomycin (15140122, Thermo Fisher Scientific). Cells were used for proliferation assay at 2 days in vitro. To assess cell proliferation, WST-1 reagent (Roche Applied Science, Indianapolis, IN), a tetrazolium salt, was added to the medium and incubated for 1 h at 37 °C in 5% CO_2_. After WST-1 incubation with cells, bathing media from 24-well plates was transferred into 96-plates and cell proliferation was determined by measuring the absorbance at 460 nm (reference wavelength 600 nm) for cleavage of the tetrazolium salt to formazan. A portion of the wells were used to evaluate the number of cells at the beginning of the experiment (t0 value), by adding WST-1 directly to the medium of 3 wells. Other wells were treated with tPA, vehicle or control medium by renewing medium. After 24 h of treatment, cells were incubated with WST-1 to estimate the number of cells after treatment (t1 value). Percentage of proliferation during 24 h (with or without treatment) was calculated using the following formula: [% of proliferatio*n =* ((t1 value/t0 value) × 100)-100].

### Chemokinesis assay and immunocytochemistry

OPC migration was assessed in chemotaxis chambers with polycarbonate membranes (pore size 8 μm; Corning Costar). The membranes were coated with poly-L-lysine (0.1 mg/mL) and laminin (0.1 mg/mL) (23017015, Thermo Fisher Scientific) as described previously [19]. OPCs from rat were seeded (40000 cells/transwell) in the upper chamber while in the lower compartment the DMEM culture medium containing N2 supplement, 2 mM glutamine, penicillin streptomycin was supplemented for the different experimental groups as follows: Control; FGF2 (0.2 μg/mL; RD Systems 233-FB); tPA 0.1, 1, 10 μg/mL (Actilyse; BoehringerIngelheim); tPA buffer (vehicle); tPA 10 μg/mL + inhibitor of EGFR kinase, AG1478 (5 μM; 1276, Tocris); AG1478 (5 μM); tPA GGACK 10 μg/mL. Concerning experimental groups, tPA 10 μg/mL + AG1478 5 μM and AG1478 5 μM, the cells were also treated one hour before and during the experiment with the EGFR blocker AG1478 5 μM and the rest of the cultures were exposed to an equal volume of the vehicle DMSO (Sigma-Aldrich-Aldrich) during the course of the experiment which was carried out at 37 °C, 5% CO_2_, and at 95% relative humidity. After 24 h, cells were fixed with 4% paraformaldehyde (PFA; for 10 min at RT), washed 3 times with phosphate buffer saline (PBS, pH 7.4) and the non-migratory cells on the upper membrane surface were removed with a cotton swab. The presence of transmigrated OPCs in the lower chamber was evaluated by immunostaining with Olig2 antibody (1:200, AB9610 Millipore) and its corresponding fluorescent secondary antibody. After immunostaining, the Boyden filters were examined and images were digitally captured using a Leica DM6000 microscope-coupled coolsnap camera and visualized with Metamorph 5.0 software (Molecular Devices). To quantify chemokinesis, 16 fields per well (×20 objective) taken randomly were photographed and the number of transmigrated OPCs per field was counted using ImageJ 1.49e software (NIH). The data were expressed as number of migrating OPCs per mm^2^ ± standard error to the mean (SEM).

### Production of proteolytically inactive tPA (GGACK tPA)

GGACK (1,5-dansyl-L-glutamyl-L-glycyl-L-arginine chloromethylketone; EMD) was added to Actilyse (BoehringerIngelheim) in a fourfold molar excess. The solution was allowed to react for 24 h at room temperature and dialysed for 48 h at 4 °C with PBS to remove all unbound GGACK. The actilyse buffer was reconstituted with arginine monohydrochloride (Sigma-Aldrich-Aldrich) added to GGACK-tPA. Finally, the lack of proteolytic activity of GGACK-tPA was confirmed with a spectrozyme assay (American Diagnostica).

### Immunoblotting

After dissociation with ice-cold TNT buffer (50 mM Tris–HCl pH 7.4; 150 mM NaCl; 0.5% Triton X-100), cells were centrifuged (12,000 g, 4 °C, 15 min) and protein content assessed by the BCA method (Pierce, France). Proteins (20 μg) were separated by 10% SDS-PAGE and transferred onto a PVDF membrane. Membranes were blocked with TBS (10 mM Tris; 200 mM NaCl; pH 7.4) containing 0.05% Tween-20, 5% BSA, and incubated overnight at 4 °C with primary antibodies against Erk 1/2 (42 kDA) and phosphorylated Erk 1/2 (42 kDA) (Cell signaling). After incubation with the anti-rabbit peroxydase-conjugated secondary antibodies (1:50000), proteins were visualized with an enhanced chemiluminescence ECL-Plus detection system (Perkin ElmerNEN, France).

### Statistical analysis

All results are expressed as mean ± SEM. For in vitro experiments, the n value corresponds to n different well pools derived from independent dissections. For group comparison, Kruskal-Wallis tests were used followed by Mann–Whitney U-tests as post hoc tests.

## Results

### Migration of OPC is delayed in tPA^−/−^ mice during spinal cord and corpus callosum development

**Fig. 1:**
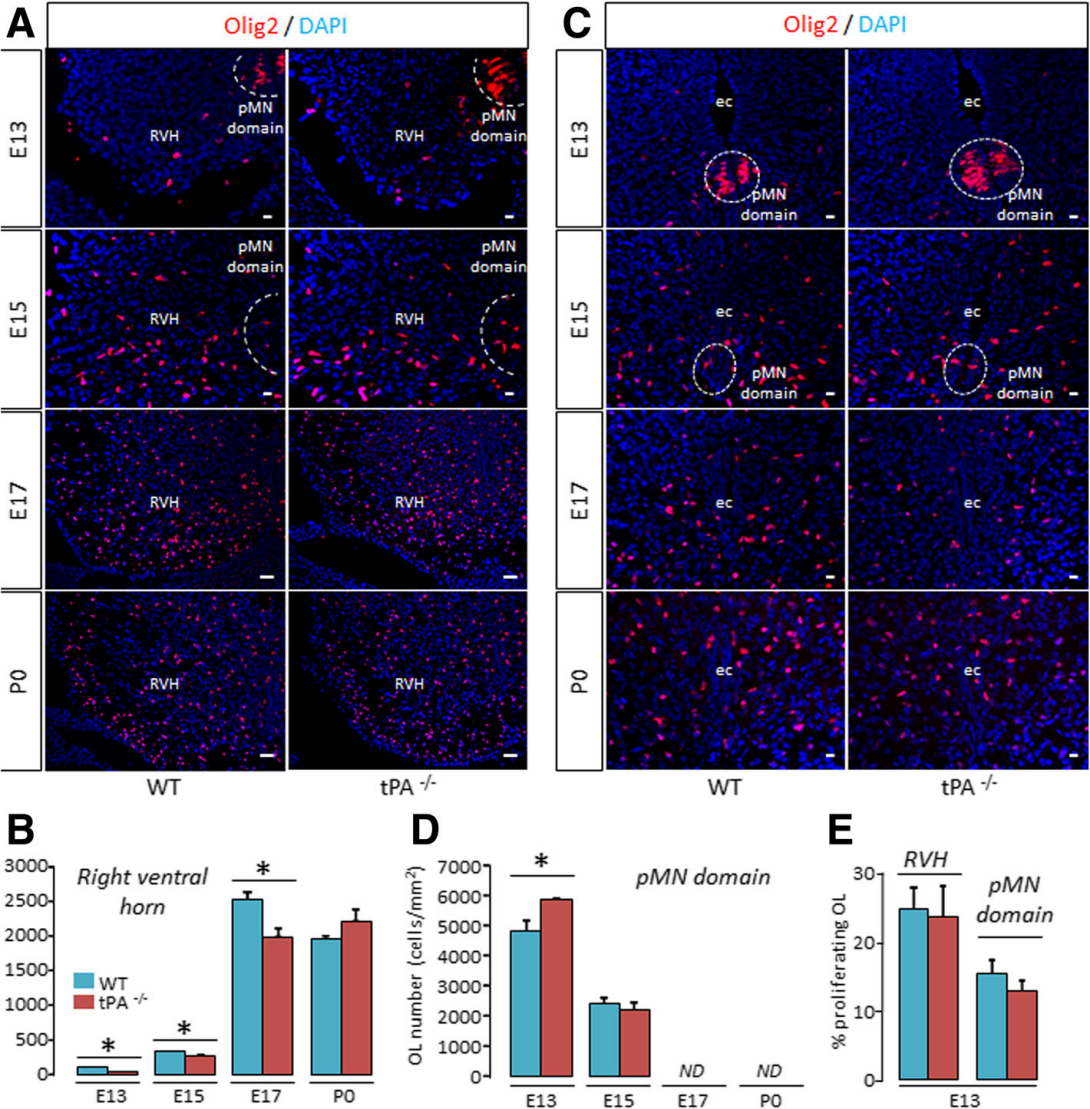
*Migration of OPC is delayed in tPA* ^*−/−*^ *mice during spinal cord development*. **a** Photomicrographs from spinal cord sections show representative images (from 3–4 mice) of Olig2 immunostaining (*red*) and DAPI staining (*blue*) in the spinal cord (*right ventral horn*) of WT (left column) and tPA^−/−^ (*right column*) mice at E13, E15, E17 and P0. **b** Corresponding quantifications of Olig2^+^ oligodendrocytes (mean ± SEM, *n =* 3–4 per group; *p < 0.05). **c** Photomicrographs from tissue sections show representative images (from 3–4 mice) of Olig2 immunostaining (*red*) and DAPI staining (*blue*) in pMN domain of WT (*left column*) and tPA^−/−^ (*right column*) mice at E13, E15, E17 and P0. **d** Corresponding quantifications of Olig2^+^ oligodendrocytes (mean ± SEM, *n =* 3–4 per group; *p < 0.05). **e** Quantification of the percentage of proliferating OLs (Ki67^+^/Olig2^+^) in the spinal cord parenchyma (*right ventral horn* and *pMN domain*) (mean ± SEM, *n =* 3–4 per group). *E: embryonic day; ec: ependymal canal; ND: not detected; OL: oligodendrocyte; P: postnatal day; pMN domain: motor neuron progenitor domain; RVH: right ventral horn; WT: wild type. tPA* ^*−/−*^ *: tPA Knock-out*. Scale bars: 20 μm

**Fig. 2:**
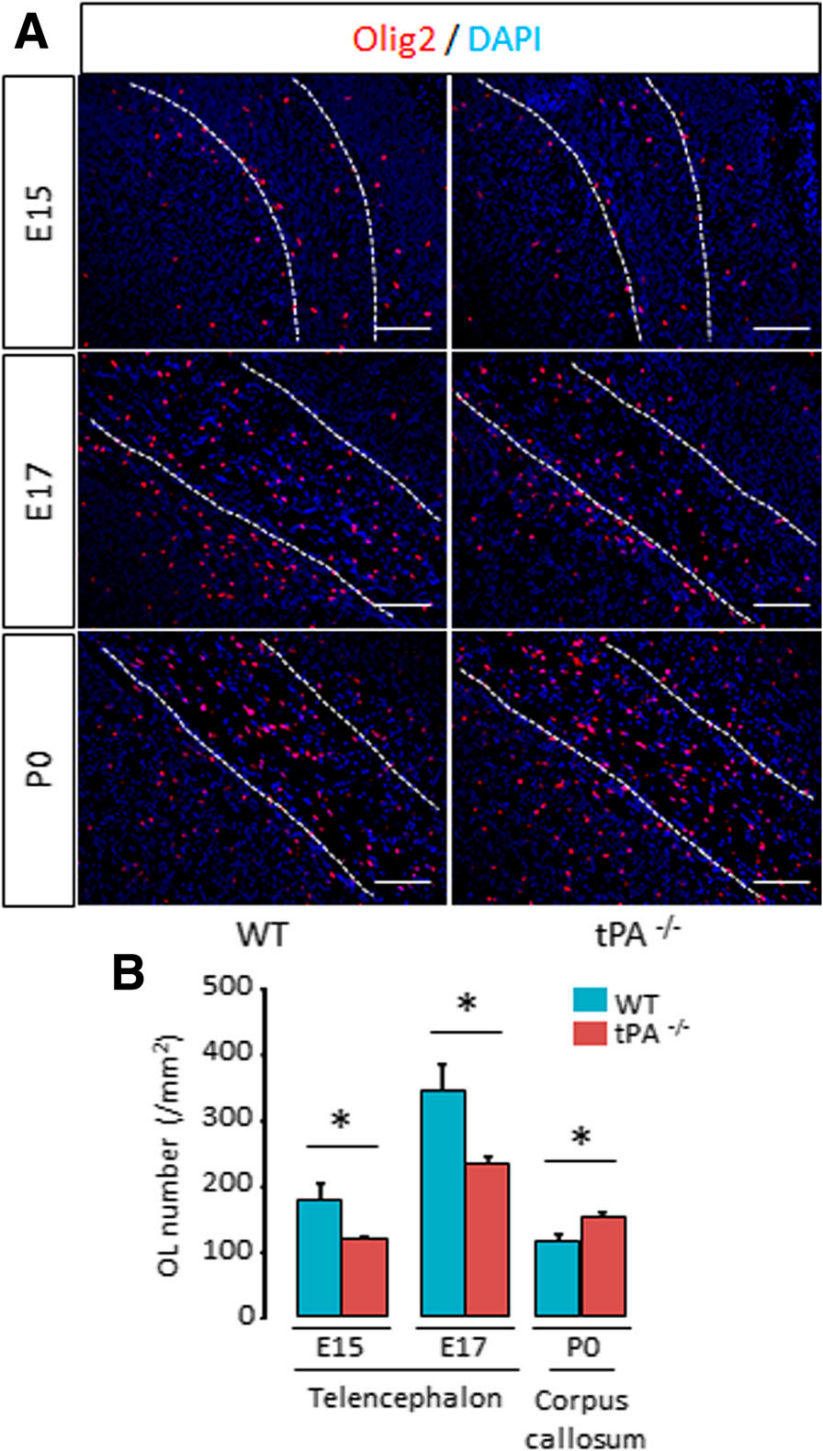
*Migration of OPC is delayed in tPA* ^*−/−*^ *mice during corpus callosum development*. **a** Photomicrographs from tissue sections show representative images (from 3 mice) of Olig2 immunostaining (*red*) and DAPI staining (*blue*) in the telencephalon of WT (*left column*) and tPA^−/−^ (*right column*) mice during corpus callosum development at E15, E17, P0 and P3. Dotted lines show corpus callosum limits. **b** Corresponding quantifications of Olig2^+^ oligodendrocytes (mean ± SEM, *n =* 3 per group; *p < 0.05). *E: embryonic day; OL: oligodendrocyte; P: postnatal day; WT: wild type.* Scale bars: 100 μm

Our first step was to estimate the effects of the invalidation of tPA gene on ventral OPCs migration during spinal cord development (Fig. 1). The ventral OPCs originate from the motor neuron progenitor (pMN) domain that first produces motor neurons followed by OPCs [20]. We observed a lower number of Olig2^+^ cells in the spinal cord parenchyma, outside the pMN domain, in tPA^−/−^ mice than in wild type mice at E13 (WT: 117.61 ± 5.34 vs tPA^−/−^: 44.90 ± 8.87, *p =* 0.0339), E15 (WT: 342.67 ± 5.69 vs tPA^−/−^: 276.62 ± 16.41, *p =* 0.0339) and E17 (WT: 2517.52 ± 107.76 vs tPA^−/−^: 1990.55 ± 109.47, *p =* 0.0209) (Fig. 1a and corresponding quantifications, Fig. 1b). There was no significant difference between wild type and tPA^−/−^ mice at P0 (WT: 1957.72 ± 46.20 vs tPA^−/−^: 2216.869 ± 167.66, *p =* 0.1489) (Fig. 1a and corresponding quantifications, Fig. 1b). Conversely, a higher number of Olig2^+^ OPCs remained in the pMN domain, in tPA^−/−^ mice than in wild type mice at E13 (WT: 4854.75 ± 345.08 vs tPA^−/−^: 5889.12 ± 30.20, *p =* 0.0339) (Fig. 1c and corresponding quantification, Fig. 1d). There was no significant difference between wild type and tPA^−/−^ mice at E15 (WT: 2632.84 ± 192.33 vs tPA^−/−^: 2230.11 ± 202.74, *p =* 0.4795). Olig2^+^ cells were not detected inside the pMN domain at E17 and P0, (Fig. 1c and corresponding quantifications, Fig. 1d). This effect was not due to an action of tPA on OPC proliferation, as the number of Ki67^+^/Olig2^+^ proliferating OPCs was not altered in tPA^−/−^ mice, either within or outside the pMN at E13 (Fig. 1e). These data suggest that the lower number of OPCs outside the pMN observed in the spinal cord of tPA^−/−^ mice is due to a delay in the migration ability of OPCs. We then asked whether this effect of tPA is restricted to the spinal cord or could also be important for the development of other brain regions. To answer this, we used the same strategy as before to assess OPC migration in the telencephalon of mice between E15 and P0 (Fig. 2), at stages of corpus callosum formation. In accordance with what observed in the spinal cord, we observed a lower number of Olig2^+^ cells in the telencephalon parenchyma in tPA^−/−^ mice as compared to wild type mice at E15 (WT: 179.21 ± 22.48 vs tPA^−/−^: 120.54 ± 2.49, p = 0.0495) and E17 (WT: 342.73 ± 42.56 vs tPA^−/−^: 232.22 ± 13.35, p = 0.0495) (Fig. 2a and corresponding quantifications, Fig. 2b). At P0, this wave of OPC migration stopped, as reflected by a decrease in the number of Olig2^+^ both in wild type and tPA^−/−^ animals. This trend occurred with a delay in tPA^−/−^, resulting in a higher number of Olig2^+^ cells in tPA^−/−^ at P0 (WT: 114.82 ± 9.48 vs tPA^−/−^: 151.13 ± 9.20 p = 0.0495). Together, these data show that tPA deletion leads to a delay in the migration of OPCs during the formation of the spinal cord and the corpus callosum.

**Fig. 3:**
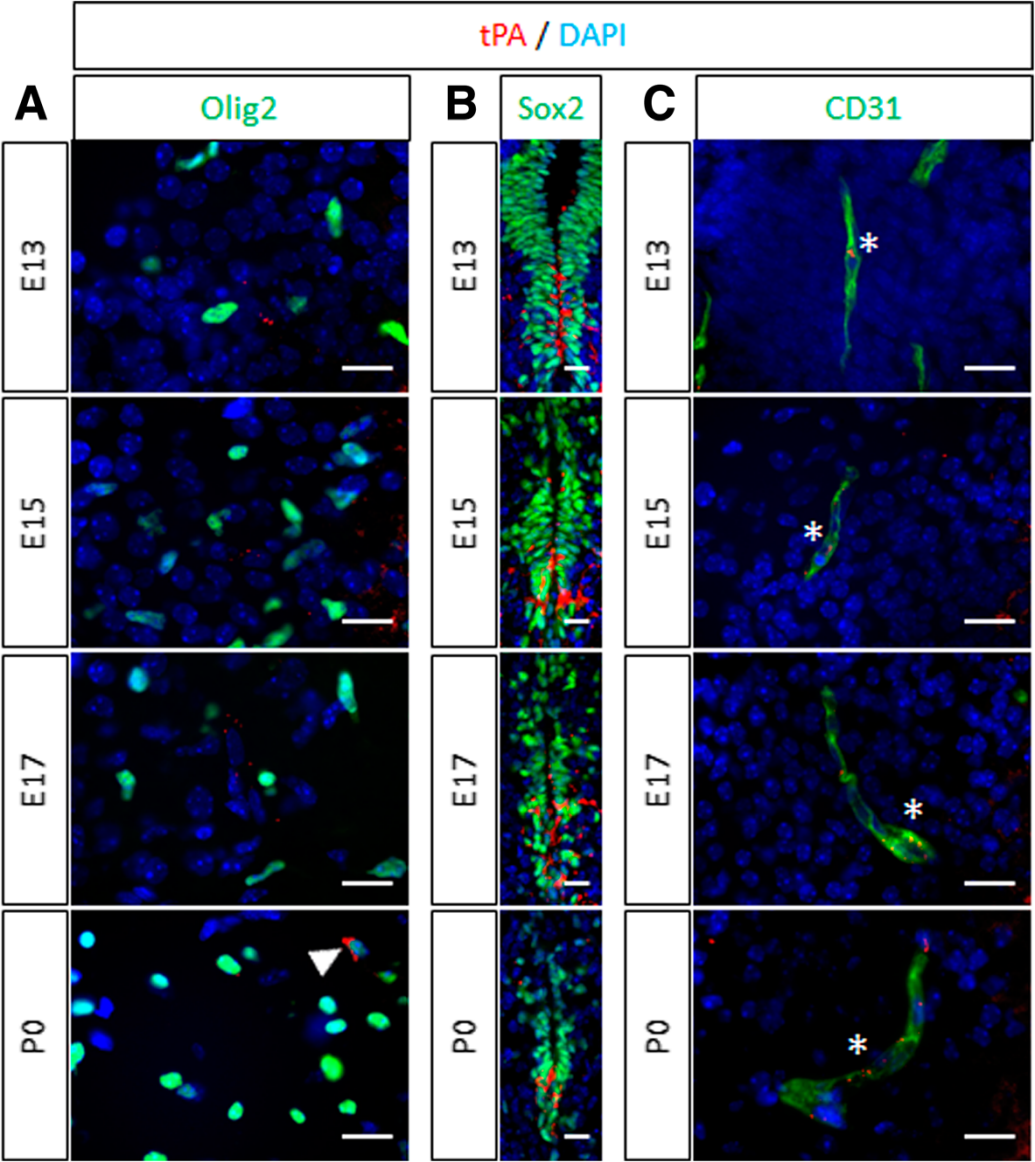
*tPA expression during spinal cord development*. Photomicrographs from spinal cord sections show representative images (from 3 mice) of tPA (*red*) with (**a**) Olig2 (*green*), (**b**) Sox2 (*green*), and (**c**) CD31 (*right column, green*) immunostaining in the spinal cord of wild type mice at E13, E15, E17 and P0. Asterisks show colocalization of tPA and CD31 immunoreactivity in endothelial cells. Filled arrowhead shows colocalization of tPA and Olig2 immunoreactivity oligodendrocytes. *E: embryonic day; P: postnatal day*. Scale bars: 20 μm

**Fig. 4:**
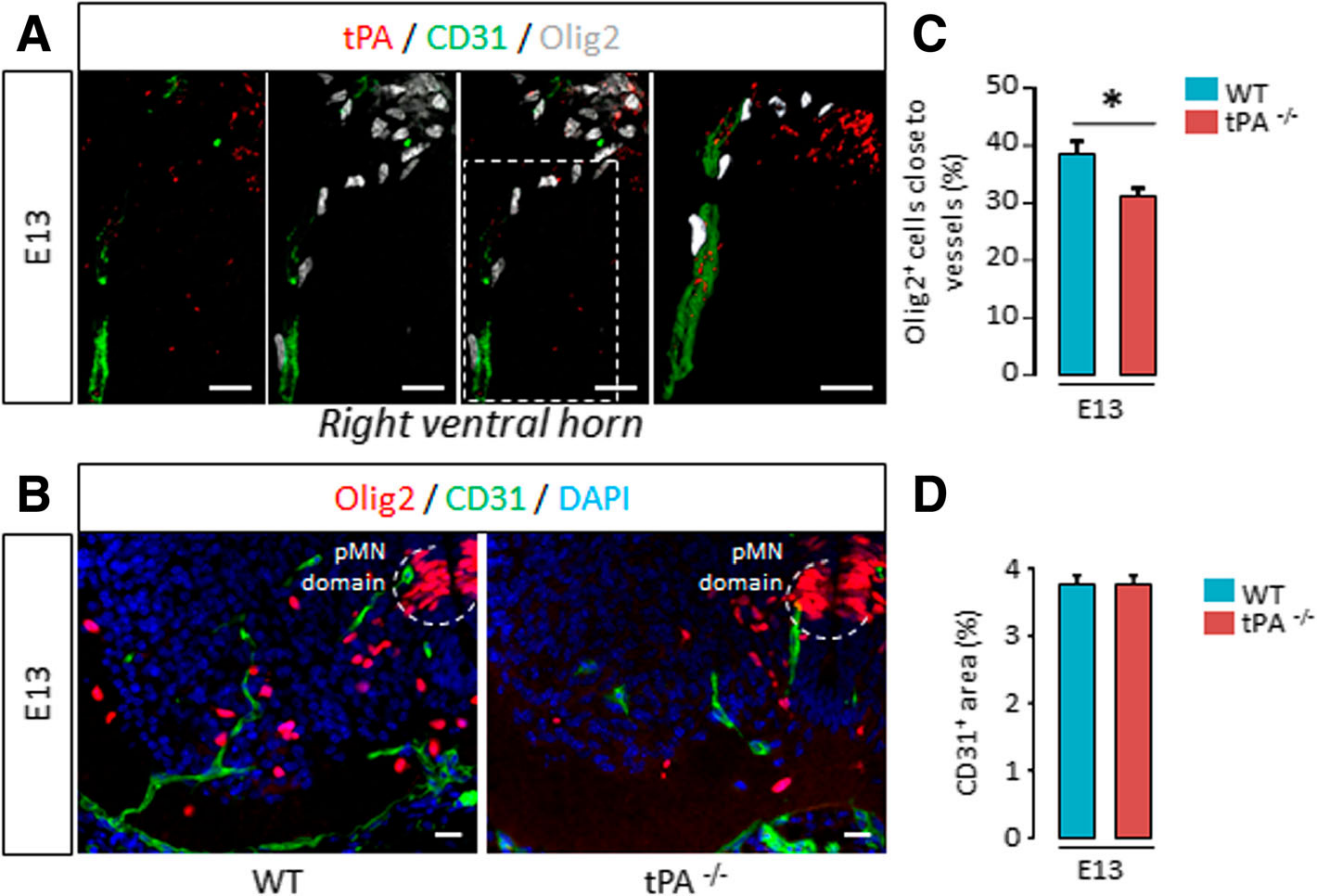
*tPA promotes oligodendrocyte migration along vessels during development at times of intense migration*. **a** Photomicrographs from spinal sections show representative images (from 3 mice) of tPA (*red*), CD31 (*green*) and Olig2 (*grey*) immunostaining in the right ventral horn of WT mice spinal cords at E13. Right image is a 3D reconstruction from confocal acquisitions (on the left). **b** Photomicrographs from tissue sections show representative images (from 3 mice per group) of Olig2 (*red*), CD31 (*green*), and DAPI (*blue*) immunostaining in the right ventral horn of WT and tPA^−/−^ mice spinal cords at E13. **c** Quantification of the percentage of Olig2^+^ oligodendrocytes close (<10 μm) to vessels in the ventral spinal cord parenchyma outside the pMN domain in WT and tPA^−/−^ mice (mean ± SEM, *n =* 3 per group; *p < 0.05). **d** Quantification of the percentage of CD31^+^ staining area in the ventral spinal cord parenchyma outside the pMN domain in WT and tPA^−/−^ mice (mean ± SEM, *n =* 3 per group; *p < 0.05). *E: embryonic day; OL: oligodendrocyte; pMN domain: motor neuron progenitor domain; WT: wild type.* Scale bars: 20 μm

Our next step was to determine the cellular origin of tPA during development in wild type mice (Fig. 3). tPA expression in mature oligodendrocytes did not occur before P0 (Fig. 3a), confirming previous reports in adult mice [11]. However, tPA was expressed in Sox2^+^ neural stem cells of the pMN (Fig. 3b) and in CD31^+^ endothelial cells (Fig. 3c) within the whole spinal cord from the early stages of spinal cord development (E13) to P0. Then, because OPC migration has been shown to be guided by endothelial cells during development [21], we hypothesized that invalidation of tPA may result in loss of OPC tropism for endothelial cells. We focused on embryonic day 13 to study oligovascular interactions because E13 signs the beginning of the first wave of OPC migration in the mouse spinal cord [22] and because the biggest difference in oligodendrocyte migration between wild type and tPA^−/−^ were observed at this stage (Fig. 1a-b). First, in the right ventral horn of spinal cord in wild type mice, we observed that a substantial proportion of Olig2^+^ cells (Fig. 4a) was found at the vicinity (<10 μm) of vessels at E13. In tPA^−/−^ mice, we found significantly less OPCs at the vicinity of vessels than in wild type mice (WT: 38.86 ± 2.02 vs tPA^−/−^: 31.33 ± 1.25, *p =* 0.0495) (Fig. 4b, and corresponding quantification Fig. 4c). Of note, the number of vessels was not altered in tPA^−/−^ mice as indicated by quantification of the surface occupied by CD31 immunostaining (WT: 3.76 ± 015 vs tPA^−/−^: 3.79 ± 0.13, *p =* 0.8273) (Fig. 4d).

### Migration of OPC is delayed in tPA^−/−^ mice during remyelination after white matter lesion in adult mice

**Fig. 5:**
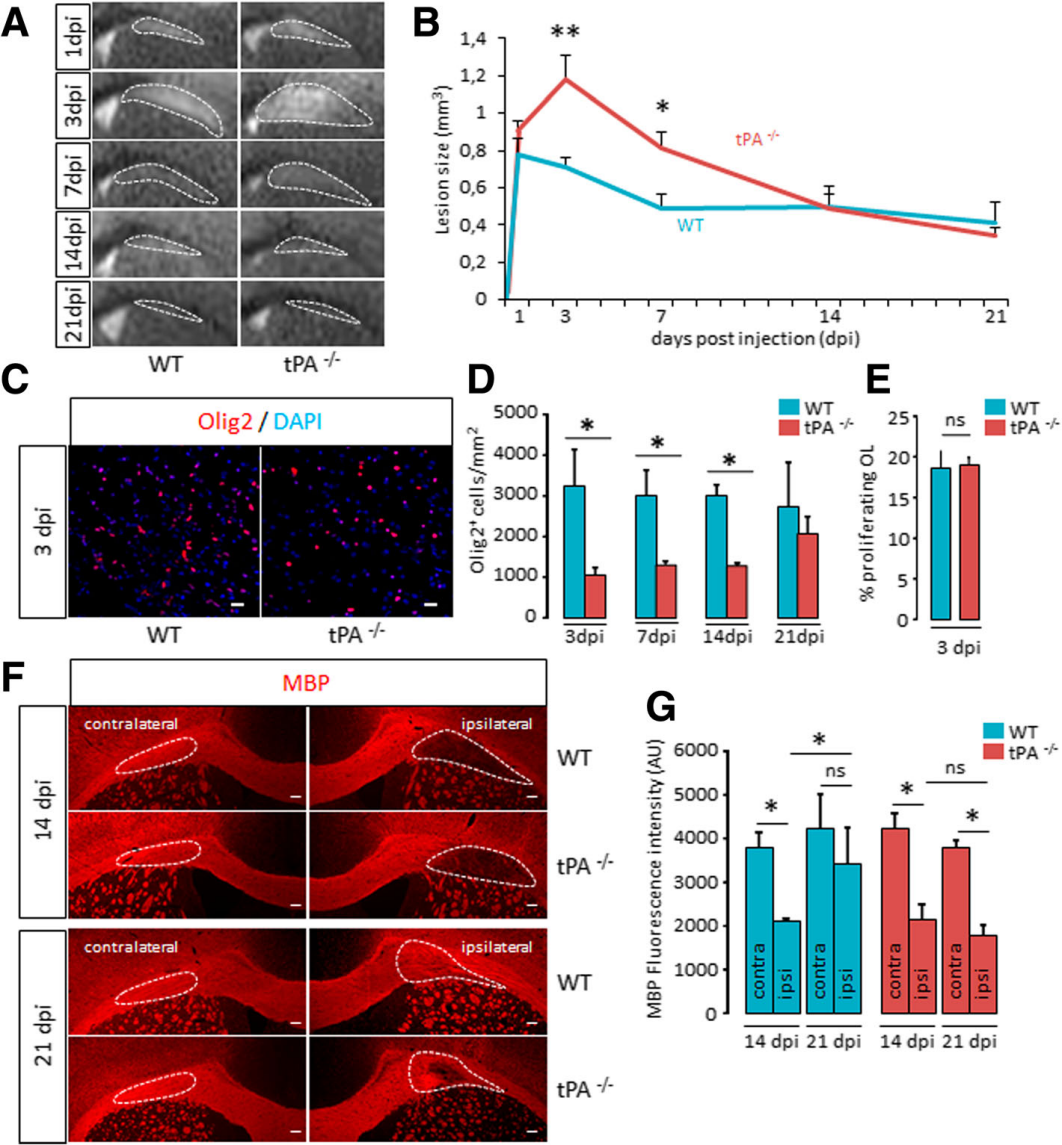
*Remyelination is delayed in tPA* ^*−/−*^ *after white matter damage in adult mice*. **a** Representative high resolution T2 images and **b** quantification of the lesion size at 1, 3, 7, 14 and 21 days after lysolecithin injection (dpi) in the corpus callosum of WT and tPA^−/−^ mice. (mean ± SEM, *n =* 5 per group; *p < 0.05 and **p < 0.01 significantly, Mann–Whitney *U* test). **c** Photomicrographs from corpus callosum sections (perilesion) show representative images (from 3 mice) of Olig2 immunostaining (red) and DAPI staining (blue) from WT (left) and tPA^−/−^ (*right*) mice at 3 days post injection (dpi). (Scale bars: 20 μm). **d** Corresponding quantifications of Olig2^+^ oligodendrocytes (mean ± SEM, *n =* 3 per group; *p < 0.05). **e** Quantification of the percentage of proliferating OLs (Ki67^+^Olig2^+^/Olig2^+^) in the lesion of WT and tPA^−/−^ mice, 3 days post-injection. (mean ± SEM, *n =* 3 per group). **f** Photomicrographs from brain sections (corpus callosum lesion) show representative images (from 3 mice per group) of MBP immunostaining (*red*) in the ipsilateral and contralateral corpus callosum of WT and tPA^−/−^ mice, 14 and 21 days post-injection (dpi). Dotted lines show quantified area limits. (Scale bars: 100 μm). **g** Corresponding quantifications of MBP fluorescence intensity (mean ± SEM, *n =* 3 per group; *p < 0.05). *AU: arbitrary unit; dpi: days post injection; OL: oligodendrocyte; WT: wild type. tPA* ^*−/−*^ *: tPA Knock-out*

Because remyelination after lesion of the white matter shares mechanistic features with myelination during development [9, 10], we then addressed the possible involvement of tPA in remyelination. To do so, we induced focal white matter lesions in adult mice by stereotactic injection of lysolecithin directly in the corpus callosum [23]. As seen with MRI analysis, this induced the formation of a white matter lesion (Fig. 5a), followed by progressive recovery along time from 3 to 21 days post injection (dpi) (Fig. 5a and corresponding quantification, Fig. 5b). At 3 dpi, tPA^−/−^ presented larger lesions than wild type mice (WT: 0.71 ± 0.05 mm^3^ vs tPA^−/−^: 1.18 ± 0.13 mm^3^, *p =* 0.009) (Fig. 5a and corresponding quantification, Fig. 5b). However, at 1dpi, lesion size was equivalent in wild type and tPA^−/−^ animals (WT: 0.78 mm^3^ ± 0.09 vs tPA^−/−^: 0.91 ± 0.05 mm^3^, *p =* 0.1745) (Fig. 5a and corresponding quantification, Fig. 5b) which indicates that the larger lesions in tPA^−/−^ at 3 dpi are due to a prolonged progression of the lesion. In fact, at 3 dpi, recovery had already started in wild type animals, while the lesion continued growing in tPA^−/−^ animals (Fig. 5a and corresponding quantification, Fig. 5b). White matter lesions were still larger in tPA^−/−^ at 7 dpi (WT: 0.49 mm^3^ ± 0.08 vs tPA^−/−^: 0.82 mm^3^ ± 0.09, *p =* 0.0283) (Fig. 5a and corresponding quantification, Fig. 5b), to eventually reach equivalent size a 14 dpi (WT: 0.5 ± 0.07 vs tPA^−/−^: 0.49 ± 0.12, *p =* 0.9168). This suggests a delayed recovery in tPA^−/−^ animals, which could be due to a defect in OPC migration. Indeed, we observed that the number of Olig2^+^ cells was lower in tPA^−/−^ at 3dpi, 7dpi and 14 dpi, and reached equivalent values at 21dpi (WT: 0.41 ± 0.11 vs tPA^−/−^: 0.34 ± 0.04, *p =* 0.7540) (Fig. 5c and corresponding quantification, Fig. 5d). This effect was not due to an action of tPA on OPC proliferation, as the number of Ki67^+^/Olig2^+^ proliferating OPCs was not altered in tPA^−/−^ at 3dpi (Fig. 5e). No difference was observed in the number of Olig2^+^ cells in the contralateral corpus callosum at these times (Additional file 2: Figure S1). We asked whether these differences in the number of Olig2^+^ cells within the lesion could result in differences in remyelination. At 14 dpi, wild type and tPA^−/−^ corpus callosum showed comparable MBP staining with a remaining lesion in the ipsilateral corpus callosum (WT: 2110.94 ± 79.37 vs tPA^−/−^: 2143.10 ± 354.94, *p =* 0.8273) (Fig. 5f and corresponding quantification, Fig. 5g), indicating that remyelination was incomplete in wild type and tPA^−/−^ animals at this stage. At 21dpi, in wild type animals, MBP staining increased in the ipsilateral to reach an intensity close to the contralateral, unlesioned hemisphere, and the lesion mostly disappeared (Fig. 5f and corresponding quantification of MBP staining, Fig. 5g), which indicates effective remyelination between 14 and 21 dpi. In contrast, in tPA^−/−^ animals, MBP staining did not increase between 14 and 21 dpi and a lesion remained in the corpus callosum (Fig. 5f and corresponding quantification of MBP staining, Fig. 5g), which indicates that remyelination was mostly ineffective between 14 and 21 dpi. Together, these data show that the delay in OPC migration in tPA^−/−^ is associated to a defect in remyelination.

**Fig. 6:**
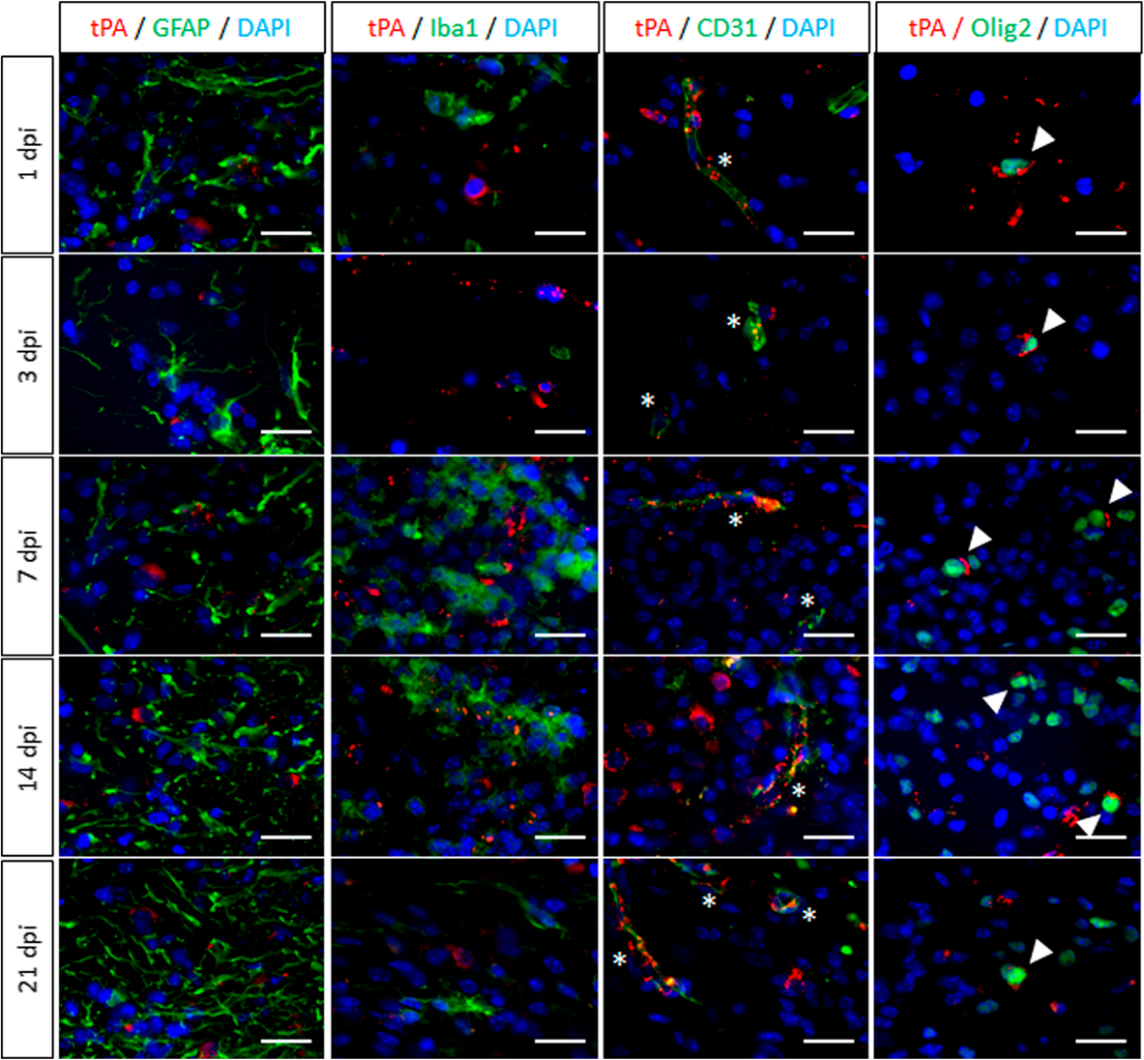
*tPA expression after white matter damage in wild type mice*. Photomicrographs from tissue sections show representative images of tPA (*red*), GFAP (*green, first column*), Iba1 (*green, second column*), CD31 (*green, third column*), Olig2 (*green, fourth column*) and DAPI staining (*blue*) immunoreactivities in the remyelinating corpus callosum of WT mice, 1, 3, 7, 14 and 21 days after lysolecithin injection. Asterisks show colocalization of tPA and CD31 immunoreactivities in endothelial cells. Filled arrowheads show colocalization of tPA and Olig2 immunoreactivities in oligogendrocytes. (Representative images, *n =* 3 per group). *dpi: days post injection*. Scale bars: 20 μm

**Fig. 7:**
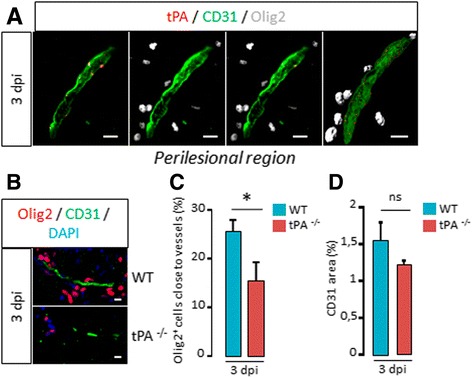
*tPA promotes oligodendrocyte migration along vessels after white matter damage*. **a** Photomicrographs from brain sections show representative images (from 3 mice) of tPA (*red*), CD31 (*green*) and Olig2 (*grey*) immunostaining in the corpus callosum lesion of WT mice at 3 days post injection (3 dpi). Right image is a rotation of 3D reconstruction from confocal acquisitions (on the *left*). **b** Photomicrographs from tissue sections show representative images (from 3 mice per group) of Olig2 (*red*), CD31 (*green*), and DAPI (*blue*) immunostaining in the corpus callosum (perilesion) of WT and tPA^−/−^ mice spinal cords at 3 days post injection (3 dpi). **c** Quantification of the percentage of Olig2^+^ oligodendrocytes close (<10 μm) to vessels in the in the corpus callosum (perilesion) in WT and tPA^−/−^ mice (mean ± SEM, *n =* 3 per group; *p < 0.05). **d** Quantification of the percentage of CD31^+^ staining area in corpus callosum (perilesion) in WT and tPA^−/−^ mice (mean ± SEM, *n =* 3 per group; *p < 0.05). *E: embryonic day; OL: oligodendrocyte; pMN domain: motor neuron progenitor domain; WT: wild type.* Scale bars: 10 μm

We next addressed the question of the cellular origin of tPA during post-lesion remyelination (Fig. 6). tPA expression was found around the lesion in wild type mice in endothelial CD31^+^ cells and in some Olig2^+^ oligodendrocytes at all tested stages (1–21 dpi), but not in Iba1^+^ or GFAP^+^ cells (Fig. 6). Similar to what previously observed during developmental myelination, oligodendrocyte migration took place preferentially along the vessels (Fig. 7a) at 3 dpi and this tropism of oligodendrocyte for vessels was reduced in tPA^−/−^ mice as compared to wild type mice (WT: 25.57 ± 2.39 vs tPA^−/−^: 15.51 ± 3.79, *p =* 0.0495) (Fig. 7b and corresponding quantification of MBP staining, Fig. 7c). As previously observed during developmental myelination, the area of CD31^+^ staining was equivalent in the lesion in wild type and tPA^−/−^ mice (WT: 1.52 ± 0.27 vs tPA^−/−^:1.21 ± 0.07, *p =* 0.5127) (Fig. 7d), indicating that the number of vessels was not altered in tPA^−/−^ animals.

### tPA exerts chemokinetic effects via its EGF-like domain

**Fig. 8:**
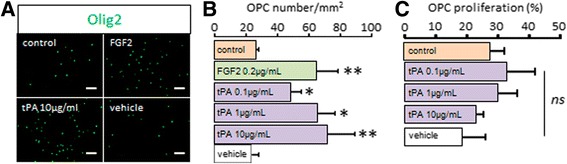
tPA induces chemokinesis on cultured OLs. **a** Rat primary cultures of OPCs were seeded on the upper compartment of Boyden chambers and treated in indicated conditions for 24 h. Photomicrographs show representative fields of the lower compartment after Olig2 immunostaining (*green*; representative image, *n =* 9, three independent cultures). **b** Oligodendrocyte chemokinesis was estimated by counting cells in the lower compartment in control conditions or in the presence of 0.2 μg/mL FGF2, 0.1-10 μg/mL tPA or tPA vehicle. (mean ± SEM; *n =* 9, three independent cultures; *p < 0.05 and **p < 0.01). **c** OL proliferation (percentage) during 24 h was estimated by measuring mitochondrial activity with WST-1 test after treatment or not. Control is the basal OL proliferation during 24 h without treatment (mean + SEM; *n =* 15, five independent cultures). *OPC: oligodendrocyte precursor cell; ns: not significant.* Scale bars: 100 μm

The above data show that the loss of tPA drives a delay in developmental myelination and in remyelination after white matter lesion. This suggests that endogenously produced tPA participates in promoting (re)myelination. To confirm that tPA could drive this effect by promoting the migration of OPCs, we used an in vitro system designed to assess chemokinesis of cultured OPCs (Fig. 8). We showed that tPA promoted the migration of OPCs in a dose dependent manner (0.1- 10 μg/ml), while tPA vehicle did not induce any effect (Fig. 8a and corresponding quantification Fig. 8b). At the highest dose (10 μg/ml), the effect of tPA on the migration of OPCs was equivalent to what observed with the chemokinetic factor FGF2 (Fig. 8a and corresponding quantification Fig. 8b). In accordance to what observed in vivo (Fig. 5e), tPA did not exert this effect by influencing OPC proliferation (Fig. 8c).

Finally, because EGFR activation in oligodendrocytes can increase OPC migration [16] and because tPA was reported earlier to activate EGFR on oligodendrocytes [11], we hypothesized that the observed effects of tPA on OPC migration implied EGFR activation.

**Fig. 9:**
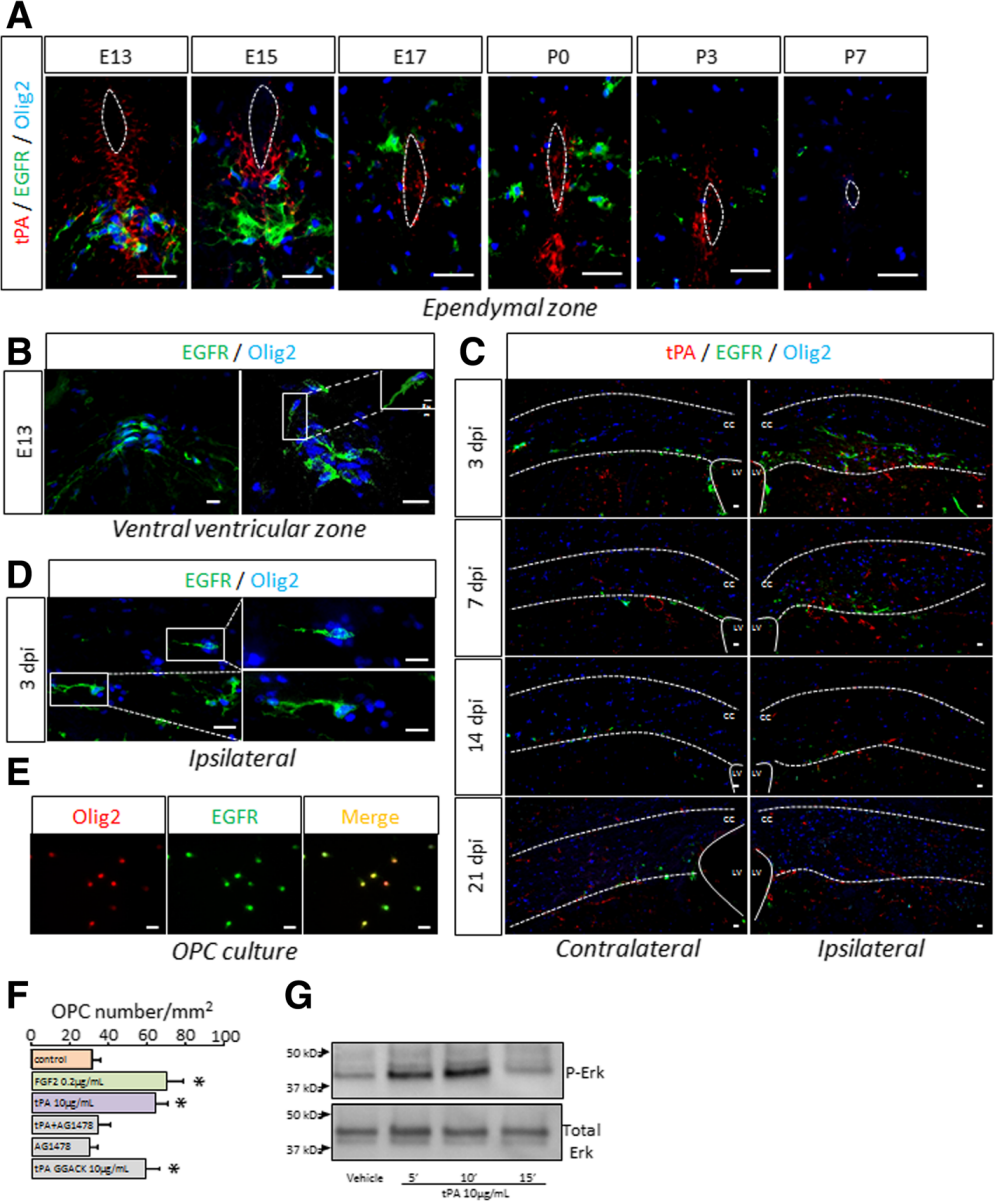
*tPA induces chemokinesis on OLs* via *its EGF-like domain*. **a** Photomicrographs from WT mice tissue sections show representative images of tPA (*red*), EGFR (*green*) and Olig2 (*blue*) immunoreactivities in the ventral ventricular zone of mice spinal cords at E13, E15, E17, P0, P3 and P7. Dotted lines show ependymal canal limits. (Representative images, *n =* 3 per group). **b** Photomicrographs from WT mice tissue sections show representative images of EGFR (*green*) and Olig2 (*blue*) immunoreactivities in the pMN domain of mice spinal cords at E13. The inlet shows 3D reconstruction from confocal acquisition. (Representative images, *n =* 3 per group). **c** Photomicrographs from WT mice tissue sections show representative images of tPA (*red*), EGFR (*green*) and Olig2 (*blue*) immunoreactivities in the contralateral (*left column*) and ipsilateral (*right column*) corpus callosum of adult mice, 3, 7, 14 and 21 days after lysolecithin injection. Full lines show lateral ventricle limits and dotted lines show corpus callosum limits. (Representative images, *n =* 3 per group). **d** Photomicrographs from adult WT mice tissue sections show representative images of EGFR (*green*) and Olig2 (*blue*) immunoreactivities in the lesion 3 days after injection. (Representative images, *n =* 3 per group). **e** Rat OPCs were cultured for 24 h. Photomicrographs show representative fields after Olig2 (*red*) and EGFR (*green*) immunostaining or the merged images (*yellow*). (representative images, *n =* 3 per group). **f** Rat primary cultures of OPCs were seeded on the upper compartment of Boyden chambers and treated in indicated conditions for 24 h. **g** Proteins from cultured OPCs treated with tPA (10μg/ml) or corresponding vehicle for indicated times were subjected to western blot for the phosphorylated (P-Erk) or total (Total Erk) forms of Erk. Chemokinesis was estimated by counting cells in the lower compartment in control conditions or in the presence of 0.2 μg/mL FGF2, 10 μg/mL tPA, 10 μg/mL tPA + 5 μM inhibitor of the kinase activity of EGFR (AG1478), 5 μM AG1478, 10 μg/mL tPA complexed with the proteolytic inhibitory peptide GGACK. (mean ± SEM; *n =* 9, three independent cultures; *p < 0.05, Mann–Whitney *U* test). *cc: corpus callosum; dpi: days post injection; E: embryonic day; LV: lateral ventricle; OPC: oligodendrocyte precursor cell; P: postnatal day.* Scale bars: 20 μm

First, we investigated whether oligodendrocytes express EGFR, which would confer them potential responsiveness to EGF-like signals mediated by tPA. During spinal cord development, EGFR was detected in the pMN domain (ventral ventricular zone) (Fig. 9a) in Olig2^+^/Sox2^+^ OPCs (Fig. 9b, Additional file 3: Figure S2) at early stages of OPC migration (E13), a subset of these cells being also positive for PDGFR-α (Additional file 3: Figure S2). In all cases, OPCs lost EGFR staining at later developmental stages, when they have left the pMN domain (Fig. 9a, Additional file 3: Figure S2). Interestingly, EGFR^+^ OPCs were found at the vicinity of tPA immunoreactivity within the pMN domain (Fig. 9a), which was compatible with an interaction of tPA with EGFR on OPCs. Concerning remyelination after white matter lesion, EGFR was expressed in the damaged corpus callosum from 3dpi, and this expression gradually disappeared with time until 21dpi (Fig. 9c). Consistent with a role of EGFR in the migration of OPCs, this receptor was detected, in the remyelinating lesion, in Olig2^+^/Sox2^+^ cells (Fig. 9d, Additional file 3: Figure S2). Together, these data indicate that EGFR, in the conditions studied here, is expressed in early, Olig2+/Sox2+ oligodendrocyte precursors.

Our last step was to address the implication of EGFR in tPA effects in our chemokinesis assay in vitro. We confirmed that cultured OPCs express EGFR (Fig. 9e). As described before (Fig. 8), tPA (10 μg/ml) induced a chemokinetic effect equivalent to the typical chemokine FGF2 (Fig. 9f). Noteworthy, while the EGFR inhibitor AG1478, when added alone, did not induce any effect on OPC migration, it reversed the chemokinetic effect of tPA (Fig. 9f). In contrast, OPC migration was not modified when the enzymatic activity of tPA was blocked by the inhibitory peptide GGACK (Fig. 9f). Finally, tPA treatment in OPC induced an early (from 5 min) and transient phosphorylation of the extracellular-regulated kinases (Erk), a downstream effectors of the EGFR pathway (Fig. 9g), as reported earlier [11]. Together, these data show that tPA can promote chemokinetic effects on OPCs via the protease-independent activation of EGFR.

## Discussion

The present study reports the effects of tPA on the migration of OPCs during embryonic development and during remyelination after white matter lesion. We show that, in both situations, OPC migration is delayed in tPA^−/−^ animals. This was illustrated by a lower number of OPCs leaving the pMN zone (spinal cord development) or reaching the remyelinating corpus callosum (post-lesion recovery) in tPA^−/−^ mice. During development and regeneration, we observed that tPA was expressed in vessels. Interestingly, in both cases, OPC migration occurred along vessels and the proportion of OPCs surrounding these vessels was reduced in tPA^−/−^ animals. Finally, we highlighted the role of EGFR signaling in these processes by showing that EGFR was expressed on migrating OPCs and that, in vitro, tPA exerted a chemokinetic effect on OPCs which was mediated by the activation EGFR.

The involvement of tPA in the migration of neurons during development of the CNS has been first shown more than 15 years ago [14] and has been confirmed later [24]. The present study brings new advances by showing that oligodendrocyte migration is also facilitated by tPA. In fact, tPA increases the migration of several other cell types, within or outside the central and peripheral nervous system, such as macrophages [25, 26] or cancer cells [27]. However, in the CNS, while most studies report the influence of tPA on migration during development, the issue of how the pro-migratory effects of tPA can influence pathological situations has been scarcely addressed [28]. Our study brings new elements by highlighting the potential role of tPA on oligodendrocyte migration during white matter damage. This suggests implication for several pathologies in which white matter is damaged, such as multiple sclerosis, leukodystrophies, periventricular leukomalacia, white matter stroke, head trauma or spinal cord injury.

Interestingly, in our study, we show that tPA invalidation leads to a delay in oligodendrocyte migration during development and after white matter demyelination. This suggests that, despite the fact that the oligodendrocyte populations involved in these two situations are different [29], their chemokinetic response to tPA would be equivalent. Pro-migratory effect of tPA was observed in the corpus callosum and in the spinal cord, which suggests equivalent responses in these two regions of the CNS. Thus, tPA appears to rise homogeneous responses in oligodendrocytes migration regardless of the age (embryo, adult), region (telencephalon, spinal cord) or pathological state.

Although the mechanism of action of tPA on migration was suggested before to involve proteolytic degradation of the extracellular matrix [24], our data show that tPA can also exert protease independent chemokinetic effects on OPCs by activating the EGFR. Several previous in vitro studies reported protease-independent effects of tPA on neurons [30–32] and oligodendrocytes precursors [11] via a mechanism termed as cytokine- or growth factor–like. In OPCs, EGF receptor activation by tPA was shown to mediate antiapoptotic effects, thus sustaining protection of the white matter after experimental ischemic stroke [11]. The present study indicates that tPA can also exert pro-migratory effect on OPCs by activating EGFR. Several differences in the experimental approach explain why pro-migratory, rather than anti-apoptotic effects are unveiled in the present work. First, the lesion model used here is based on the detergent properties of lysolecithin which toxic effects largely supplants apoptosis. In effect, the lesion size at 1dpi is mostly similar in wild type and tPA^−/−^ animals, and the effect of tPA invalidation appears only from 3dpi, when OPC migration and remyelination starts. Second, our study addresses the effect of endogenous tPA, while in previous works [11] tPA was injected to the animals. The impact of tPA injection largely differs from that of endogenous tPA in terms of dose, timing and accessibility to injured tissues. Finally, the timing in which the study was conducted is also different: while in the Correa et al. paper, the impact of tPA injection was assessed in the subacute phase (1 day post ischemia), in the present study, histological analysis were performed in subacute and chronic phases (up to 21dpi), which enables observing tissue regeneration, in particular OPC migration.

The activation of EGFR pathway has been previously described as an activator of OPC migration. Migratory NG2^+^ cells express more EGFR than non-migratory cells [16] and overexpression in non-migratory cells prompts them to migrate [16]. Accordingly, EGFR-expressing retinal progenitor cells show increased chemokinesis in the presence of EGF [33]. In the context of white matter lesion, overexpression or invalidation of EGFR signaling in oligodendrocytes respectively accelerate [34] or reduces [35] remyelination after focal demyelination of corpus callosum. In light of these findings, the effect of intranasal EGF treatment was tested in a mouse model of preterm diffuse white matter injury [36]. This treatment led to enhanced generation of OPCs, leading to functional recovery [36]. Chemokinetic effects were proposed to sustain these effects of EGFR signaling activation, as EGF induces neural precursor cells to differentiate to glial cells and to acquire a motile phenotype [37]. In our study, we suggest that this EGFR-dependent pathway could be activated by tPA to exert pro-migratory effects on OPCs in pathological conditions where white matter is damaged, without inducing hyperplasia of OPCs as reported when EGFR is constitutively expressed [38].

The increase in the number of OPCs during remyelination upon EGFR activation has been suggested in the past to occur via increased proliferation, enhanced differentiation from neural progenitors, migration or combinations of these different mechanisms [39–41]. Here we show that tPA does not influence OPC proliferation in vitro, but rather enhances their migration via a chemokinetic dependent on the activation of EGFR. One possibility to explain this difference is that the activation of EGFR by tPA, by its natural ligand (EGF) or by genetic strategies can induce different response in target cells, in particular because the degree of activation of downstream targets in these different situations may vary considerably.

Our in vitro data suggest that tPA mediates its promigratory effects, at least in part, by activation of EGFR and independently of its proteolytic activity. Nevertheless, proteolytic effects have also been suggested: tPA was shown to proteolytically activate proBDNF into mature BDNF, which increases proliferation of OPCs in vitro [42] andpromotes remyelination when injected intra veinously after WM stroke [43]. In our hands, tPA invalidation did not modify proliferation of OPC, which claims for a different mechanism. Activation of growth factor pathways by tPA can also occur by a regulation of expression, rather than maturation: tPA upregulates vascular endothelial growth factor (VEGF) by endothelial cells [44] which others have reported to promote the migration of oligodendrocyte precursor cells in vitro [45, 46]. Notheworthy, whether the VEGF pathway targets OPC proliferation [47] or not [46] is still a matter of debate. In any case, we report here that tPA deficiency did not influence OPC proliferation. Further studies may help decipher possible links between tPA and the VEGF pathway in OPC migration in vivo, during development and/or remyelination.

The previous point highlights the possible cooperative role between endothelial cells and OPCs during (re)myelination. This has led to the concept of « oligovascular niche » [48]: vascular cells would secrete soluble factors (including VEGF) which promotes migration and survival of oligodendrocytes [49]. Because tPA is mainly produced by endothelial cells, it could be one of these oligotrophic factors. In our hands, the common cellular source of tPA in development and adult remyelination was endothelial cells, which corroborates a possible role of endothelial-derived tPA in the observed effects on OPC migration. This is particularly relevant to the increasing literature describing that migration of progenitor cells, including OPCs, is guided by vessels. Blood vessels form a scaffold for migration of neuroblasts to the adult olfactory bulb [50], and more recently, a similar mechanism has been described specifically for OPCs migration [21]. A cooperation between vessels and progenitors exists, in which neural progenitors (including OPCs) induce local angiogenesis while migrating, which in turns facilitates progenitor emigration from the niche [51]. This « vasophilic » migration [50] was described during development in previous studies, but the present work is to our knowledge the first to describe it during remyelination after white matter lesion. This is particularly interesting in regard to the description of perivascular tPA deposits in acute MS lesions [52], a type of demyelinated plaque where remyelination may succeed spontaneously.

Pericytes have also been suggested to influence OPC migration [53], which raises the question of the expression of tPA in pericytes. During development, we did not detect pericytes in the developing spinal cord at E13, when the phenotypic differences between wt and tPA^−/−^ first occurred (data not shown), which is likely due to the immaturity of the vessels of the spinal cord at this stage, in which pericytes are usually not reported [54–56]. After white matter lesion, tPA was not detected in pericytes surrounding blood vessels at 3 dpi (Additional file 4: Figure S3), when the delay in OPC migration was observed in tPA^−/−^ as compared to wt mice. This is in accordance with our previous report of the absence of tPA in adult pericytes [12]. Nevertheless, we not cannot exclude with certitude that tPA is not expressed in pericytes, and this should be the purpose of future studies.

This link between angiogenesis and OPC migration is relevant to white matter pathology, in particular multiple sclerosis, in which angiogenesis has been described [57]. tPA could be involved in these processes in regard to a previous study in experimental autoimmune encephalomyelitis, a model of multiple sclerosis, in which recovery is reduced in tPA^−/−^ animals [58]. Noteworthy, while wild type animals showed remyelination in late phases of the disease, this remyelination was severely reduced in tPA^−/−^ animals [58]. The present study brings new elements in a model where demyelination is the primary insult:we here show that the delay in OPC migration to the damaged area results in reduced remyelination in tPA^−/−^ mice. This strengthens the idea that the failure in recovery in tPA^−/−^ in EAE could be the result of a reduced capacity of remyelination. Overall, the present data suggest that endogenous tPA could be important for remyelination in multiple sclerosis. In accordance with this, tPA activity has been shown to be decreased in post-mortem tissues from MS patients [59], alleging that a drop of tPA may participate in reducing the opportunity of remyelination in MS patients.

## Conclusion

The present study shows that tPA provides chemokinetic effects on OPCs. This effect leads to facilitating migration of these cells during CNS and remyelination after white matter lesion. Our in vitro results suggest that this effect is mediated by the activation of EGFR, expressed on OPCs, by the virtue of the EGF-like domain contained in tPA structure. These results highlight the potential role of tPA on OPCs in situation of white matter lesion. They suggest that, in combination with previously described anti-apoptotic activity, the chemokinetic effects of tPA could be targeted to improve myelin recovery in pathological conditions.

## Additional files

Additional file 1: Table S1. Antibodies used in the study. Each antibody used in this study is listed together with its supplier, species, type (monoclonal/polyclonal), dilution and reference. (DOCX 16 kb)
Additional file 2: Figure S1.
*Oligodendrocyte number in the contralateral corpus callosum of tPA* ^*−/−*^ *and WT mice*. Quantification of OLs (Olig2^+^) in the contralateral corpus callosum of WT and tPA^−/−^ mice, 3, 7, 14 and 21 days after lysolecithin injection (mean + SEM, *n =* 3 per group). *dpi: days post injection; OL: oligodendrocyte; WT: wild type.* (TIF 523 kb)
Additional file 3: Figure S2.
*EGFR is expressed in early oligodendrocyte precursors*. Photomicrographs from embryonic spinal cord (ventral ventricular zone, E13) and perilesional adult corpus callosum (3dpi) WT mice tissue sections show representative confocal images of EGFR (green), Sox2 or PDGFRα (red) and DAPI (blue) immunoreactivities. Asterisks indicate PDGFRα^+^/EGFR^−^ cells and arrowheads indicate PDGFRα^+^/EGFR^+^ cells (Representative images from *n =* 3). Scale bars: 10 μm. (TIF 585 kb)
Additional file 4: Figure S3.
*tPA is not detected in PDFGR-β* ^*+*^ *pericytes.* (A) Photomicrographs from adult WT mice tissue sections show representative confocal images of PDGFR-β (green), tPA (red) and DAPI (blue) immunoreactivities in the lesion 3 days after injection. (Representative images from *n =* 3). Inlets show side view reconstructions of the z-stack. (B) Representative fluorescent intensity/distance graph measured from confocal image in (A) showing that tPA (Red) is not found in colocalization with PDGFR-β staining. (TIF 328 kb)

## Abbreviations

CNS: Central nervous system
dpi: Days post injection
EGF: Epidermal growth factor
MS: Multiple sclerosis
OPCs: Oligodendrocyte precursor cells
pMN: Motor neuron progenitor
tPA: Tissue-type plasminogen activator
tPA^−/−^: tPA Knock-out
WT: wild type
