# Supplementary material for: Tissue-type plasminogen activator exerts EGF-like chemokinetic effects on oligodendrocytes in white matter (re)myelination
Source: Mol Neurodegener. 2017 Feb 23;12:20. doi: 10.1186/s13024-017-0160-5 (PMC5322587; doi:10.1186/s13024-017-0160-5)
Supplement: Additional file 1: Table S1. — Antibodies used in the study. Each antibody used in this study is listed together with its supplier, species, type (monoclonal/polyclonal), dilution and reference. (DOCX 16 kb) [file 13024_2017_160_MOESM1_ESM.docx]

| Primary antibodies | Supplier | Species | Type | Dilution | Reference |
| --- | --- | --- | --- | --- | --- |
| CD31 | BD Pharmigen^TM^ | Rat | monoclonal | 1/2000 | 553370 |
| EGFR | Abcam | Rabbit | monoclonal | 1/3000 | ab52894 |
| GFAP | Abcam | Chicken | polyclonal | 1/800 | ab4674 |
| Iba1 | Wako | Rabbit | polyclonal | 1/1000 | 019-19741 |
| Ki67 | Abcam | Rabbit | polyclonal | 1/1000 | ab15580 |
| MBP | Abcam | Rabbit | polyclonal | 1/800 | ab40390 |
| Olig2 | Millipore | Rabbit | polyclonal | 1/800 | AB9610 |
| Olig2 AF®488 conjugate | Millipore | Mouse | monoclonal | 1/3000 | MABN50A4 |
| PDGFRα | BD Pharmigen^TM^ | Rat | monoclonal | 1/800 | 558774 |
| Sox 2 | Abcam | Rabbit | monoclonal | 1/1000 | ab92494 |
| Sox 2 | Santa Cruz Biotechnology | Goat | polyclonal | 1/500 | sc-17320 |
| tPA | Molecular innovations | Sheep | polyclonal | 1/1500 | SASMTPA-GF-HT |
| Secondary antibodies (Jackson Immunoresearch) | | Species | Type | Dilution | Reference |
| Cy™3 AffiniPureF(ab')₂ Fragment Donkey Anti-Rabbit IgG (H+L) | | Donkey | polyclonal | 1/600 | 711-166-152 |
| Fluorescein (FITC) AffiniPureF(ab')₂ Fragment Donkey Anti-Chicken IgY (IgG) (H+L) | | Donkey | polyclonal | 1/600 | 703-096-155 |
| Fluorescein (FITC) AffiniPure F(ab')₂ Fragment Donkey Anti-Goat IgG (H+L) | | Donkey | polyclonal | 1/600 | 705-096-147 |
| Fluorescein (FITC) AffiniPureF(ab')₂ Fragment Donkey Anti-Mouse IgG (H+L) | | Donkey | polyclonal | 1/600 | 715-096-150 |
| Fluorescein (FITC) AffiniPureF(ab')₂ Fragment Donkey Anti-Rabbit IgG (H+L) | | Donkey | polyclonal | 1/600 | 711-096-152 |
| Fluorescein (FITC) AffiniPureF(ab')₂ Fragment Donkey Anti-Rat IgG (H+L) | | Donkey | polyclonal | 1/600 | 712-096-150 |
| Rhodamine (TRITC) AffiniPureF(ab')₂ Fragment Donkey Anti-Sheep IgG (H+L) | | Donkey | polyclonal | 1/600 | 713-026-147 |
| Alexa Fluor® 647 AffiniPure F(ab')₂ Fragment Donkey Anti-Rabbit IgG (H+L) | | Donkey | polyclonal | 1/600 | 711-606-152 |

**Supplementary Table 1**
