# Supplementary figures and images for: Tissue-type plasminogen activator exerts EGF-like chemokinetic effects on oligodendrocytes in white matter (re)myelination
Source: Mol Neurodegener. 2017 Feb 23;12:20. doi: 10.1186/s13024-017-0160-5 (PMC5322587; doi:10.1186/s13024-017-0160-5)

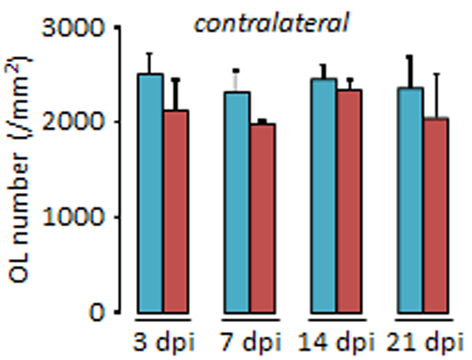

Supplement: Additional file 2: Figure S1. — Oligodendrocyte number in the contralateral corpus callosum of tPA −/− and WT mice. Quantification of OLs (Olig2+) in the contralateral corpus callosum of WT and tPA−/− mice, 3, 7, 14 and 21 days after lysolecithin injection (mean + SEM, n = 3 per group). dpi: days post injection; OL: oligodendrocyte; WT: wild type. (TIF 523 kb) [file 13024_2017_160_MOESM2_ESM.tif]

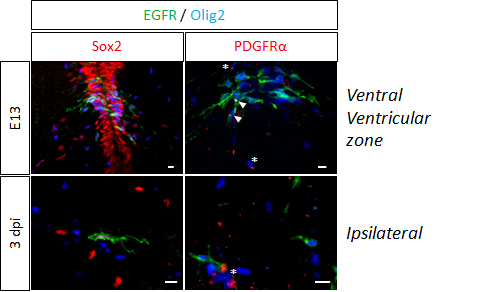

Supplement: Additional file 3: Figure S2. — EGFR is expressed in early oligodendrocyte precursors. Photomicrographs from embryonic spinal cord (ventral ventricular zone, E13) and perilesional adult corpus callosum (3dpi) WT mice tissue sections show representative confocal images of EGFR (green), Sox2 or PDGFRα (red) and DAPI (blue) immunoreactivities. Asterisks indicate PDGFRα+/EGFR− cells and arrowheads indicate PDGFRα+/EGFR+ cells (Representative images from n = 3). Scale bars: 10 μm. (TIF 585 kb) [file 13024_2017_160_MOESM3_ESM.tif]

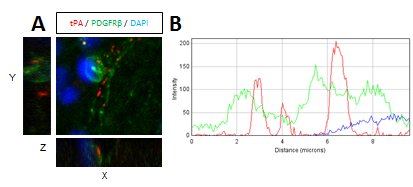

Supplement: Additional file 4: Figure S3. — tPA is not detected in PDFGR-β + pericytes. (A) Photomicrographs from adult WT mice tissue sections show representative confocal images of PDGFR-β (green), tPA (red) and DAPI (blue) immunoreactivities in the lesion 3 days after injection. (Representative images from n = 3). Inlets show side view reconstructions of the z-stack. (B) Representative fluorescent intensity/distance graph measured from confocal image in (A) showing that tPA (Red) is not found in colocalization with PDGFR-β staining. (TIF 328 kb) [file 13024_2017_160_MOESM4_ESM.tif]
